# The effect of an mLearning application on nurses’ and midwives’ knowledge and skills for the management of postpartum hemorrhage and neonatal resuscitation: pre–post intervention study

**DOI:** 10.1186/s12960-021-00559-2

**Published:** 2021-01-26

**Authors:** Aurore Nishimwe, Latifat Ibisomi, Marc Nyssen, Daphney Nozizwe Conco

**Affiliations:** 1 School of Public Health, Faculty of Health Sciences, University of the Witwatersrand, 1 Smuts Avenue, Braamfontein, 2000 South Africa; 2 School of Health Sciences/College of Medicine and Health Sciences, University of Rwanda, P.O. Box 3286, Kigali, Rwanda; 3 Nigerian Institute of Medical Research, 6 Edmund Cres, Yaba, Lagos, Nigeria; 4 Department of Biomedical Statistics and Informatics, Vrije Universiteit Brussel, Brussels, Belgium

**Keywords:** Postpartum hemorrhage, Neonatal resuscitation, mHealth, Safe delivery application, Rwanda

## Abstract

**Background:**

Globally, mobile learning (mLearning) tools have attracted considerable attention as a means of continuous training for healthcare workers. Rwanda like other low-resource settings with scarce in-service training opportunities requires innovative approaches that adapt technology to context to improve healthcare workers’ knowledge and skills. One such innovation is the safe delivery application (SDA), a smartphone mLearning application for Basic Emergency Obstetric and Neonatal Care (BEmONC) content. This study assessed the effect of the SDA intervention on nurses’ and midwives’ knowledge and skills for the management of postpartum hemorrhage (PPH) and neonatal resuscitation (NR).

**Methods:**

The study used a pre–post test design to compare knowledge and skills of nurses and midwives in the management of PPH and NR at two measurement points: immediately prior to SDA intervention and after 6 months of SDA intervention. The intervention took place in two district hospitals in Rwanda and included 54 participants. A paired-sample t-test was used to measure the pre–post intervention, mean knowledge and skills scores differences. Confidence intervals (CIs) and effect size were calculated. A t-test and a one-way Anova were used to test for potential confounders.

**Results:**

The analysis included 54 participants. Knowledge scores and skills scores on PPH management and NR increased significantly from baseline to endline measurements. The mean difference for PPH knowledge is 17.1 out of 100; 95% CI 14.69 to 19.49 and 2.6% for PPH skills; 95% CI 1.01 to 4.25. The mean difference for NR knowledge is 19.1 out of 100; 95% CI 16.31 to 21.76 and 5.5% for NR skills; 95% CI 3.66 to 7.41. Increases were unaffected by participants’ attendance to in-service training 6 months prior and during SDA intervention and previous smartphone use. However, pre- and post-intervention skills scores were significantly different by years of experience in obstetric care.

**Conclusion:**

The SDA intervention improved the knowledge and skills of nurses and midwives on the management of PPH and NR as long as 6 months after SDA introduction. The results are highly relevant in low-income countries like Rwanda, where quality of delivery care is challenged by a lack of in-service continuous training for healthcare providers.

## Background

According to WHO, Global numbers from the year 2017 estimates show that 295,000 women die during pregnancy and childbirth [1]. It is also estimated that 2 million stillbirths and 2.5 million early newborn deaths occur each year worldwide [2, 3]. More than 90% of these deaths happen in low- and middle-income countries, like Rwanda [4]. Leading experts state that between 70 and 88% maternal and newborn deaths are preventable or have treatable causes [5–8]. In Rwanda, the most frequent birth complications are postpartum hemorrhage (71.6%) and newborn asphyxia and their complications (38%) [9]. Strategies for prevention and treatment of birth complications have been proposed by the WHO, UNICEF, and UNFPA in a set of seven signal functions referred to as Basic Emergency Obstetric and Newborn Care (BEmONC). The BEmONC services are mostly provided by nurses and midwives in low-resource settings [10].

Nurses and midwives, particularly in sub-Saharan hospitals, are the key clinicians in birth care. But, substandard performance of nurses and midwives in managing birth complications has been documented widely as a major cause of maternal and newborn deaths in hospital settings globally [11]. Several studies have documented that knowledge and skills of nurses and midwives in low-resources settings are not always at an acceptable level despite their previous education [10–16]. Hence, improving competency of nurses and midwives could improve maternal and newborn outcomes. One of the approaches to improve competency of nurses and midwives is to conduct regular in-service training courses. Hosey et al. (2016) documented that promoting continuous professional development of midwives presents a constant promise of more effective and safe birth care in low-resources settings [17]. However, different logistical and financial barriers can make this approach unachievable [18, 20]. Given the accessibility of mobile phones in Africa, it is suggested that mLearning applications that provide essential obstetric training through instructional videos and self-directed learning options, such as the Safe Delivery mHealth Application (SDA), can overcome these barriers and improve birth care outcomes [19–22].

A number of studies have investigated the effectiveness of mobile health (mHealth) interventions in maternal and newborn health initiatives in low-income countries [23, 24]. For instance, an mLearning pilot study about delivering family planning refresher training using interactive voice response and text messages revealed a substantial increase in knowledge of contraceptive side effects among nurses and midwives in Senegal [25]. On the other hand, Tomlinson el al. (2013) highlighted the fact that the enthusiasm for effective mHealth interventions in sub-Saharan Africa is high, but little is known about their efficacy or effectiveness in resource-limited settings [26]. Also, most of the studies examining mLearning tools are of poor methodological quality [21]. There is limited evidence on the contribution of mLearning applications on healthcare workers’ knowledge and skills particularly in low-income countries [27]. Hence, there is a need for evidence of mLearning applications benefits as means of continuous training without interrupting healthcare services. Therefore, this study assessed the effects of the SDA mLearning application on nurses’ and midwives’ knowledge and skills in the management of the most frequent birth-related complications of postpartum hemorrhage (PPH) and newborn asphyxia in Rwanda.

The SDA is a BEmONC mLearning smartphone application developed by the Maternity Foundation, University of Copenhagen, and the University of Southern Denmark [28]. The SDA was designed to reinforce the professional competences of skilled birth attendants on how to manage BEmONC by means of an mLearning platform made of animated instructional videos and a self-explanatory learning platform called “MyLearning”. The videos last about 7 to 15 min and can be viewed either in full or by chapter. While, the learning platform allows the user to enter into his/her personalized learning journey and further improve his/her knowledge of the content provided on the SDA. The learning platform offers an opportunity to the user to test his or her knowledge using the MyLearning questionnaires. The learner moves from the beginner, intermediate, expert levels and reaches the Safe Delivery Champion level and gain a certificate as he/she passes the quizzes. We investigated whether Rwandan nurses and midwives’ use of SDA had an effect on their knowledge and skills with regards to PPH management and NR.

## Methods

### Study setting

The study took place in two district hospitals in Rwanda: Masaka hospital in Kigali, an urban province; and Nyamata hospital located in the eastern rural province [29]. The two hospitals were selected out of 12 district hospitals in the two provinces because both had a high number of deliveries per year [30]. The two hospitals have been offering BEmONC services for more than 5 years.

### Study participants

Nurses and midwives working in the maternity departments of the selected hospitals, who deal with birth care were invited to participate in the study. The inclusion criteria for nurses and midwives were set as follows: having a work experience of at least 6 months in obstetric care, being full-time employed in the selected hospital, and willing to participate in the study. The study participants included 33 midwives and 21 nurses.

### Study design

**Fig. 1:**
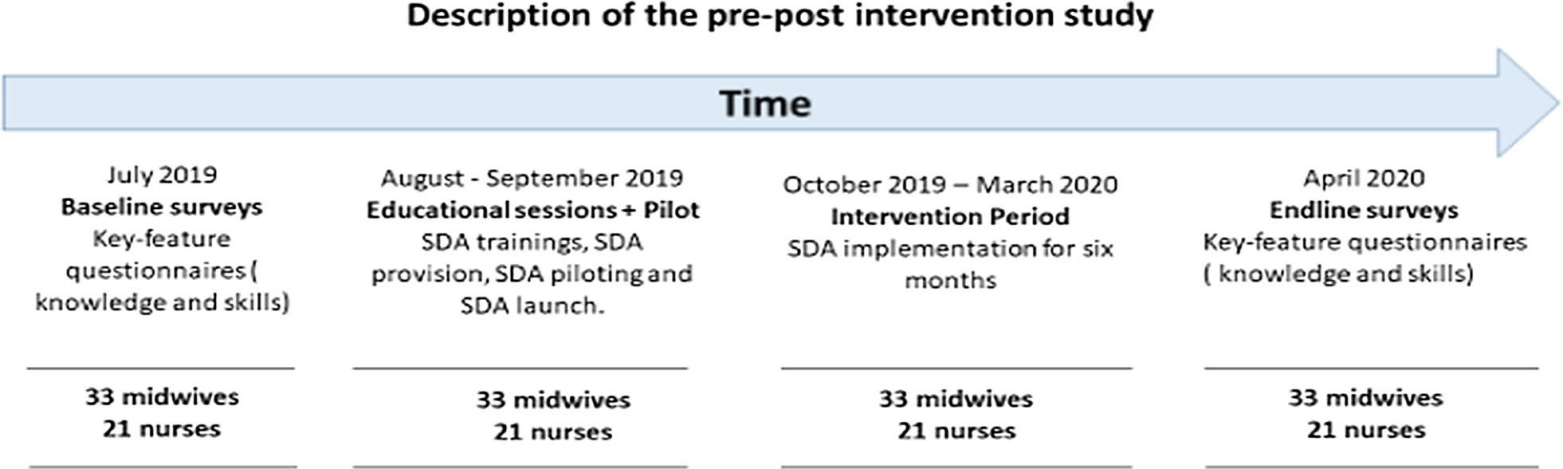
Description of the pre–post intervention study

The study adopted a pre–post test design to compare knowledge and skills of nurses and midwives in the management of PPH and NR at two measurement points: pre and post the SDA intervention. The study was carried out in four phases: establishing the baseline, the educational sessions, the intervention and the endline. The baseline and endline surveys were conducted pre and post the intervention, respectively. The 6-month intervention period was preceded by training of the participants on how to use the SDA. The study took place between July 2019 and April 2020. Figure 1 provides more details on this study design.

### Description of the SDA intervention

The SDA is an mLearning application designed to provide training for health care providers in low-income countries on how to manage obstetric and neonatal emergencies. The SDA conveys knowledge and skills, such as how to ventilate a newborn in need of resuscitation or how to remove a retained placenta by means of visual guidance using animated instructional videos and a self-explanatory learning platform. The SDA also contains a catalog with essential obstetric drugs and equipment. The content of the SDA is based on WHO clinical guidelines on BEmONC and has been validated by an international group of global health experts [31]. All features and functions in the SDA are designed for low-literacy and low-income settings and work off-line once downloaded. It can thus be adapted to local contexts and policies. The SDA can be downloaded free of charge for iPhone at https://itunes.apple.com/dk/app/safe-delivery/id985603707?mt=8 and for Android at https://play.google.com/store/apps/details?id=dk.maternity.safedelivery&hl=en.

Smartphones with the SDA were implemented at two district hospitals for a 6-month period. Nurses and midwives working in the maternity departments of the selected hospitals received a half-day SDA introduction training. The training encompassed a description of the SDA features and modules and an explanation of how to use the smartphone and SDA with joint video viewing and interactive exercises in small groups on using the SDA as a learning tool. Each of the study hospitals (Masaka and Nyamata) was allocated three smartphones, with pre-installed English and French versions of the SDA. Participants who owned smartphones, were encouraged to download the SDA for use during the training. The majority of participants (*n* = 47) downloaded the SDA on their personal smartphones for the training. During the 6-month SDA intervention, nurses and midwives used the SDA in a personalized learning journey to train their knowledge and skills on how to manage obstetric and neonatal emergencies with the assistance of the SDA installed on their personal smartphones or the smartphones provided by the researcher. The research team ensured that the provided smartphones were available to the team on duty at all times. The intervention also entailed two visits per week by the researcher and four research assistants to each of the study hospitals. During the visits, the researcher and the research assistants observed how the participants were using the SDA and encouraged them to watch the videos while they were there, they also provided guidance on how to start their learning journeys and made sure that each participant started learning for the PPH and NR modules.

### Measurement and data collection

The primary outcomes of the study were knowledge scores and skills scores of nurses and midwives. Self-administered key feature questionnaires developed and tested by Maternity Foundation were adopted, only questions regarding PPH and NR were considered. Knowledge and skills scores were measured for 2 BEmONC services, PPH management and neonatal resuscitation. The research instrument included questions on PPH management and NR knowledge and skills, demographic information, as well as exposure to in-service education on the topics under study. The knowledge questionnaire consisted of clinical content questions on causes, consequences, prevention, treatment and management of PPH and NR. The knowledge questionnaire includes 36 knowledge questions for PPH management and 37 knowledge questions for NR with single and multiple responses options. The skills were evaluated by means of a self-administered questionnaire with simulated clinical cases due to technical difficulties of evaluating skills by change in real practice. The skills questionnaire comprised six clinical scenarios simulating PPH cases for PPH management and four clinical scenarios simulating NR cases for NR with single and multiples responses options. The collected demographic data of the participants included age, gender, education level, years of experience in obstetric care, hours spent per week in providing obstetric care, and number of deliveries participated in monthly. In addition, we asked participants if they have received any in-service training about PPH management and/or neonatal resuscitation during the 6 months prior or during the SDA intervention period. This question helped the researcher to understand more about the influence of the SDA or other available in-service training. We also asked participants if they have previously used smartphones to capture their experience with smartphones.

A total of 54 nurses and midwives completed either the English or the French form of self-administered questionnaires at baseline and at 6 months post-intervention. English and French languages are both professionally used in Rwanda. Baseline measures were taken prior to the training of nurses and midwives on the use of the SDA and included: demographic data; knowledge of nurses and midwives in the management of PPH and NR; and skills of nurses and midwives in managing PPH and NR. All data were collected on paper in a classroom setting. Six months after the SDA introduction, knowledge and skills were measured a second time in the classroom setting using the same data collection instruments.

### Data management and analysis

The data were entered and checked for errors in Microsoft Excel and subsequently transferred and analyzed in Stata version 16 (StataCorp LLC). Descriptive summary statistics were computed on demographic data including age, gender, education level, years of experience, hours spent per week in providing obstetric care, delivery participated in monthly, previous use of smartphone, and in-service training for 6 months prior and during SDA intervention. Since the study targeted all nurses and midwives working in the maternity department of the selected hospitals, all the eligible participants in the maternity department, a sample of more than 30 participants was required to support the use of parametric statistical tests.

Knowledge and skills scoring were calculated by dividing the number of questions answered correctly by the total number of questions times a hundred. Respondents who scored 50% and above were considered knowledgeable. Further, Mean scores across all knowledge and skills scores were computed. A paired-sample t-test was used to measure the pre–post intervention, mean knowledge and skills scores differences (where the dependent variable was the mean difference in change on scores for the two measurements, baseline and endline). Confidence intervals (CIs) and effect size were calculated. To test for potential confounding, using a t-test, pre–post intervention mean knowledge and skills scores were compared and between group differences were calculated to examine the role of in-service training and previous smartphone use on test scores. One-way analysis of variance (ANOVA) was used to compare means scores across the three categories of experience in obstetric care (group 1: 1 year to 5 years, group 2: 6 years to 10 years, group 3: above 10 years) for pre- and post-intervention results. Significance level was set at *p* < 0.05.

### Ethics approval

This study has been approved by the Human Research Ethics Committee of the University of the Witwatersrand (M190258) and the University of Rwanda, College of Medicine and Health Sciences’ Institutional Review Board (No. 377/CMHS IRB/2018). Written consent was obtained from each participant. Confidentiality of the participants was maintained at all times. Questionnaires were number-coded thereby keeping the identity of the participants anonymous.

## Results

### Demographic characteristics of study participants

A total of 54 participants, 33 midwives and 21 nurses completed the surveys. More than a half were from Masaka district hospital (*n* = 30, 56%), the majority were female (*n* = 33, 61%), their average age was 33 years (SD = 7.1). The level of education was predominantly the advanced diploma (A1) in midwifery with 27 of 54 (50%) participants having A1 level in midwifery. The least represented level of education was the secondary school level (A2) in nursing with only one participant with A2 level in nursing. The majority of the participants had less than 6 years of experience in obstetric care (*n* = 32, 59%), spent more than 10 h per week providing obstetric care (*n* = 40, 74%) and participated in more than 10 deliveries per month (*n* = 41, 76%). Three participants have received BEmONC in-service training during the 6 months of the SDA intervention. Four participants had never used a smartphone before the study. More details are shown in Table 1 below.

**Table 1:** Demographic characteristics of study participants (*n* = 54)

	*n* (%)
Hospital affiliation	
Masaka District Hospital	30 (56)
Nyamata District Hospital	24 (44)
Gender	
Male	21 (39)
Female	33 (61)
Education level	
Midwife A0	6 (11)
Midwife A1	27 (50)
Nurse A0	5 (9)
Nurse A1	15 (28)
Nurse A2	1 (2)
Years of experience in obstetrical care	
1–5	32 (59)
6–10	15 (28)
> 10	7 (13)
Weekly workload in obstetrical care (h)	
0–5	4 (7)
6–10	10 (19)
> 10	40 (74)
Number of deliveries past month	
0–5	8 (15)
6–10	5 (9)
> 10	41 (76)
In-service trainings past 6 months	
No	51 (94)
Yes	3 (6)
Experience with smartphone	
Tried using one	50 (93)
Never tried using one	4 (7)
Age, years, mean (SD)	33 (7.1)

% weighted percent, *SD* standard deviation

### Pre–post SDA intervention differences in knowledge scores for PPH management and NR

The mean difference in PPH knowledge from pre- to post-test was statistically significant; mean difference = 17.1% (SD = 8.8, 95% CI 14.7–19.5); *p* < 0.001. The findings suggest a large effect size for PPH knowledge scores differences, Cohen’s d score = 1.8. Similarly, the mean difference in NR knowledge from pre- to post-test was statistically significant; mean difference = 19.1% (SD = 9.9; 95% CI 16.3—21.8); *p* < 0.001. The findings suggest a large effect size for NR knowledge scores differences, Cohen’s d score = 1.7. We found a significant association between the SDA intervention and nurses’ and midwives’ knowledge on both PPH and NR knowledge, 6 months after baseline (Table 2).

**Table 2:** Differences in mean knowledge scores pre- and post-SDA intervention (*n* = 54)

	Mean, SD	95% CI	*P* value	Cohen's d
PPH knowledge scores (out of 100)				
Pre, mean (SD)	45.8 (9.2)	(43.4, 48.3)	* < .001*	1.8
Post, mean (SD)	62.9 (9.4)	(60.4, 65.5)		
Pre–post difference, mean (SD)	17.1 ( 8.8)	(14.7, 19.5)		
NR knowledge scores (out of 100)				
Pre, mean (SD)	48.6 (12.3)	(45.3, 51.9)	* < .001*	1.7
Post, mean (SD)	67.7 (10.2)	(64.9, 70.4)		
Pre–post difference, mean (SD)	19.1 ( 9.9)	(16.3, 21.8)		

*Wt.%* weighted percent, *NR* neonatal resuscitation, *PPH* postpartum hemorrhage

### Pre–post SDA intervention differences in skills scores for PPH management and NR

The mean difference in PPH skills from pre- to post-test was statistically significant; mean difference = 2.6% (SD = 5.9, 95% CI 1.1, 4.3); *p* = 0.002. The findings suggest a small effect size for PPH skills scores differences, Cohen’s d score = 0.3. Similarly, the mean difference in NR skills from pre- to post-test was statistically significant; mean difference = 5.5% (SD = 6.9; 95% CI 3.7, 7.4); *p* < 0.001. The findings suggest a medium effect size for NR skills scores differences, Cohen’s d score = 0.7. We found a significant association between the SDA intervention and nurses’ and midwives’ skills on both PPH and NR skills, 6 months after baseline (Table 3).

**Table 3:** Differences in mean skills scores pre- and post-SDA intervention (*n* = 54)

	Mean, SD	95% CI	*P* value	Cohen's d
PPH skills scores (out of 100)				
Pre, mean (SD)	63.4 (9.8)	(60.8, 66.2)	*0.002*	0.3
Post, mean (SD)	66.1 (11.2)	(63.1, 69.2)		
Pre–post difference, mean (SD)	2.6 ( 5.9)	(1.1, 4.3)		
NR skills scores (out of 100)				
Pre, mean (SD)	56.5 (7.1)	(54.6, 58.5)	* < .001*	0.7
Post, mean (SD)	62.1 (8.4)	(59.8, 64.4)		
Pre–post difference, mean (SD)	5.5( 6.9)	(3.7, 7.4)		

*Wt.%* weighted percent, *NR* neonatal resuscitation, *PPH* postpartum hemorrhage

### Potential confounders

The comparisons of PPH and NR mean knowledge and skills scores by in-services training (6 months prior the intervention and 6 months during the intervention period) across the baseline and endline groups showed that there were no significant differences along in-service training lines in the pre- and post-test for both PPH and NR (Table 4). Analysis of knowledge and skills scores by previous smartphone experience did not show a significant difference for PPH and NR scores on both pre- and post-tests (Table 4). Although participants who had smartphone experience scored slightly higher on the PPH skills post-test, there was no significant difference. Similarly, there was no significant difference in mean change in PPH and NR knowledge from pre- to post-test among those experienced with smartphones and those who had never used them. Moreover, participants were classified into three groups according to their years of experience in obstetric care: group 1 (1 year to 5 years, *n* = 32); group 2 (6 years to 10 years, *n* = 15), group 3 (above 10 years, *n* = 7). The ANOVAs indicated significant group mean differences only for the NR skills pre-test (F (2,51) = 5.31, *p* = 0.008) and for the NR skills post-test (F (2,51) = 4.83, *p* = 0.012) (Table 5). As shown in Table 6, a Tukey post hoc test revealed that pre-test NR skills scores were statistically significantly higher in the group 1 compared to the group 3 (*p* = 0.006). Similarly, post-test NR skills scores were statistically significantly higher in the group 1 compared to the group 3 (*p* = 0.011). Also, post-test NR skills scores were statistically significantly higher in the group 2 compared to the group 3 (*p* = 0.023). However, there were no statistically significant differences between the pre- and post-test NR skills scores in the group 2 compared to the group 1 (*p* = 0.641 and 0.999, respectively). Also, there were no statistically significant differences between the pre-test NR skills scores in the group 3 compared to the group 2 (*p* = 0.058).

**Table 4:** Differences in knowledge and skills scores pre- and post-intervention, analyzed by in-service training and smartphone experience

	In-service training				Smartphone experience			
	Yes	No	Mean diff	*P* value	Yes	No	Mean diff	*P* value
PPH knowledge scores (out of 100)								
Pre, mean (SD)	49.7 (2.5)	45.7 (9.4)	4	0.468	46 (9.4)	44.5 (6.1)	1.5	0.756
Post, mean (SD)	61.6 (6.4)	63.1 (9.6)	1.5	0.805	63 (9.4)	63.3 (10.9)	0.3	0.953
PPH skills scores (out of 100)								
Pre, mean (SD)	54 (13.7)	59.3 (10.9)	5.3	0.424	59.1 (11.4)	58 (7.6)	1.1	0.851
Post, mean (SD)	60.6 (10.1)	66.4 (11.3)	5.8	0.392	66.9 (11.2)	56.8 (6.4)	10.1	0.083
NR knowledge scores (out of 100)								
Pre, mean (SD)	61.3 (6.4)	47.9 (12.2)	13.4	0.065	48.7 (12.3)	48 (14.3)	0.7	0.916
Post, mean (SD)	75 (8.2)	67.2 (10.2)	7.8	0.202	67.8 (10.3)	66.5 (10.3)	1.3	0.814
NR skills scores (out of 100)								
Pre, mean (SD)	53 (10)	56.7 (6.9)	3.7	0.379	56.4 (7.2)	58.3 (6.1)	1.9	0.615
Post, mean (SD)	59 (8.5)	62.2 (8.4)	3.2	0.522	61.8 (8.7)	65.3 (3.5)	3.5	0.435

*Wt.%* weighted percent, *NR* neonatal resuscitation, *PPH* postpartum hemorrhage

**Table 5:** Differences in knowledge and skills scores pre- and post-intervention, analyzed by years of experience

	Group 1 (*n* = 32)	Group 2 (*n* = 15)	Group 3 (*n* = 7)	*Df*	*F*	*P* value
PPH Knowledge scores (out of 100)						
Pre, mean (SD)	46.8 (9.5)	46.2 (9.4)	41.1 (6.7)	(2, 51)	1.10	0.341
Post, mean (SD)	64.1 (9.4)	63.5 (10.1)	56.6 (5.9)	(2, 51)	1.96	0.151
PPH Skills scores (out of 100)						
Pre, mean (SD)	59.9 (12.2)	57.4 (10.1)	58.4 (7.7)	(2, 51)	0.27	0.767
Post, mean (SD)	66.9 (11.2)	66 (12.3)	62.6 (9.3)	(2, 51)	0.43	0.655
NR Knowledge scores (out of 100)						
Pre, mean (SD)	48.7 (13.4)	49.3 (10.7)	46.7 (11.9)	(2, 51)	0.11	0.899
Post, mean (SD)	67.8 (10.9)	68.5 (10.1)	65.6 (7.6)	(2, 51)	0.19	0.827
NR Skills scores (out of 100)						
Pre, mean (SD)	58.2 (6.7)	56.3 (6.5)	49.3 (6.2)	(2, 51)	5.31	*0.008*
Post, mean (SD)	63.4 (7.2)	63.3 (8.4)	53.4 (9.9)	(2, 51)	4.83	*0.012*

*Wt.%* weighted percent, *NR* neonatal resuscitation, *PPH* postpartum hemorrhage

**Table 6:** Paired comparisons for skills scores pre- and post-intervention, analyzed by years of experience

	*P* value for Paired Comparisons with Turkey's HSD		
	Group2 vs Group1	Group3 vs Group1	Group3 vs Group2
NR Skills scores (out of 100)			
Pre-test	0.641	*0.006*	0.058
Post-test	0.999	*0.011*	*0.023*

*Wt.%* weighted percent, *NR* neonatal resuscitation, *PPH* postpartum hemorrhage, *HSD* honestly significant test

## Discussion

The current study assessed the effect of the SDA mLearning application on nurses ‘and midwives’ knowledge and skills in postpartum hemorrhage management and neonatal resuscitation in two district hospitals in Rwanda. A statistically significant increase, of the mean knowledge scores, was found post-SDA intervention, recording 17.1% for PPH management and 19.1% for NR. The increase of the mean skills scores for PPH management and NR was also statistically significant, at 2.6% and 5.5%, respectively. The significant increase in knowledge is further supported by Cohen d large effect size. On the other hand, for skills scores, NR had a medium effect size and PPH Management had a small effect size. The difference in the effect size between knowledge and skills could be associated with measurements tools utilized to evaluate skills because we did not assess skills in real practice rather used simulated clinical cases. The analysis of knowledge and skills scores by potential confounders (in-service training 6 months prior and during intervention and smartphone experience) did not show a significant difference for PPH and NR scores on both pre- and post-tests. However, the analysis of skills scores by years of experience in obstetric care has shown a significant difference in skills scores for both pre- and post-measurements in different categories of years of experience. This study is important because it addresses the need of a potential solution to nurses and midwives’ problems of accessing up-to-date learning resources in low- and middle-income countries including Rwanda.

Our findings mirror those of other studies in two ways. First, the revelation that mHealth learning tools are essential for facilitating continuous learning and upgrading knowledge and skills of different health professional cadres is consistent with findings of a study conducted in Senegal that documented the association of a mLearning tool with sustained knowledge gains for family planning content [25]. The study highlighted the usefulness of mLearning to provide in-service training without interrupting health services [25]. Secondly, the potential of mLearning tools to improve healthcare workers’ knowledge and skills which results in improvement of maternal and neonatal healthcare services in LMICs is corroborated by systematic reviews that demonstrated the effectiveness of mHealth interventions in maternal and child healthcare [32, 33]. These findings together underline the importance of mLearning tools for facilitating in-service training particularly in low-resources settings where continuous training opportunities are scarce and the costs of training are unaffordable.

The SDA mLearning application was associated with an increase in knowledge mean scores among nurses and midwives on PPH management and NR following the 6-month SDA implementation. Likewise, a mixed-methods feasibility and pilot cluster randomized trial using the safe delivery app study conducted in the democratic republic of Congo revealed a similar increase in PPH and NR knowledge scores among health workers at 3 months post-SDA intervention [34]. Several studies have shown that mHealth interventions can improve healthcare services delivery processes which lead to improved outcomes [35–37]. A study in Tanzania assessed performance and adoption of an eLearning system utilized by healthcare providers to enhance maternal and perinatal health care for a period of 18–21 months [38]. The study reported that healthcare providers had acquired and utilized new knowledge and skills in clinical practice at 89% level at the end of the intervention [38]. Another study in Nigeria provided health care workers with mHealth tutorial application for changing the knowledge and attitude on Ebola virus disease and reported an 11% significant improvement in average knowledge levels between pre- and post-intervention scores [39]. Our findings support those of other studies [34, 38] that the use of mHealth is perceived as an opportunity for personalized continuous learning with effects on health care workers’ knowledge update. Therefore, it is important to highlight that mLearning tools such as the SDA could be considered as integrated parts of other efforts to support nurses and midwives continuous learning thus improving the quality of delivery care.

Moreover, the current study has found a significant association between the SDA mLearning application and skills of PPH management and NR among nurses and midwives. Similar findings were documented in a randomized clinical trial conducted in Ethiopia which determined the association between the safe delivery app and health care workers’ knowledge and skills in neonatal resuscitation [31]. The trial reported a more than twofold significant increase in health care workers’ skills and knowledge scores for neonatal resuscitation, 6 months following the SDA intervention [31]. It is noteworthy that in our study, improvements in average knowledge and skills of PPH management and NR occurred across all cadres irrespective of their access to other available in-service trainings. However, the years of experience played a significant role in different skills scores for both pre- and post-tests in neonatal resuscitation. This can be partly explained by the fact that nurses and midwives might have been used to routine procedures in neonatal resuscitation. The effectiveness of the SDA mLearning application in changing nurses’ and midwives’ knowledge and skills of PPH management and NR may be attributed to disseminating educational materials in real time combined with the convenience of unrestricted access to online learning materials by nurses and midwives over an unlimited time period. This underlines the potential benefits of mLearning tools to enhance nurses’ and midwives learning. mHealth tools in general present an opportunity for improving maternal health care services in underserved remote areas of low-resource settings by broadening knowledge and skills of healthcare workers.

The implications of this study are that the SDA intervention increases the knowledge and skills of nurses and midwives in managing obstetric and neonatal emergencies. Evolving efforts for continuing education in Rwanda and similar contexts should consider the integration of mLearning as an approach for training and as a job aid for BEmONC in order to reduce maternal and newborn mortality, as well as considering the integration of mLearning tools for other priority and emergent health problems. Challenges to implementation of quality BEmONC care, posed by gaps in the environmental context and resources, as well as the regulatory and accountability environment, must be considered and addressed alongside other programmatic measures such as quality improvement initiatives to target health system weaknesses.

### Strengths and limitations of this study

The study has three limitations. First, it is limited by the small sample of 54 nurses and midwives, which reflects the fact that the SDA intervention was implemented only in two district hospitals due to economic barriers. Therefore, the current study will not generalize findings. Second, we acknowledge the limitation of findings when assessing skills using a self-administered questionnaire with simulated clinical cases. This was due to technical difficulties of evaluating skills by change in real practice. Third, we acknowledge that some of the improvements in knowledge and skills observed in this study may arise from exposure to other learning materials other than the SDA during the intervention period. Also, the increase in knowledge and skills does not necessarily equal better outcomes for mothers and newborns which is the ultimate goal. The findings in the current paper are part of a large study evaluating the feasibility of the SDA in Rwanda. Findings of the effect of the SDA on maternal and newborn outcomes will be discussed in another paper.

Nevertheless, the evidence that the SDA intervention is effective in improving knowledge and skills of nurses and midwives in the management of PPH and NR sets the stage for expanding coverage and further testing of the SDA intervention to other hospitals in Rwanda. Additionally, the accuracy of our findings is increased by supporting the knowledge and skills surveys with focus group discussions with nurses and midwives, to provide context to and clarify reasons behind some participant responses. The findings of the focus group discussions will be presented in a separate paper.

## Conclusion

In conclusion, the SDA mLearning application appears to be effective for improving nurses’ and midwives’ knowledge and skills in the management of PPH and NR. The changes in knowledge and skills recorded from the evaluation of this SDA mLearning intervention suggest that mLearning tools have potential for facilitating continuous training of nurses and midwives without interrupting healthcare services. The user-friendly nature of this SDA and regular access to updated learning materials via visual guidance using animated videos and a self-explanatory learning platform, streamed in real time to nurses and midwives, is a strong selling point of this SDA mLearning application. Using mLearning tools as a means of sustaining the knowledge and skills of nurses and midwives to manage delivery complications and improving maternal and newborn healthcare in LMICs should be explored further, especially given the increased global utilization of mobile technology and the corresponding economic benefit.

## Data Availability

The dataset generated for this study will be made available from the corresponding author on a reasonable request.

## Abbreviations

BEmONC: Basic emergency obstetric and newborn care
LMICs: Low- and middle-income countries
mLearning: Mobile learning
mHealth: Mobile Health
SDA: Safe Delivery mHealth application
PPH: Postpartum hemorrhage
NR: : Neonatal resuscitation
SBAs: Skilled birth attendants
WHO: World Health Organization
UNICEF: United Nations International Children’s Emergency Fund
UNFPA: United Nations Population Fund
